# Assessing Respiratory Activity by Using IMUs: Modeling and Validation

**DOI:** 10.3390/s22062185

**Published:** 2022-03-11

**Authors:** Vito Monaco, Carolina Giustinoni, Tommaso Ciapetti, Alessandro Maselli, Cesare Stefanini

**Affiliations:** 1 The Biorobotics Institute and the Department of Excellence in Robotics and AI, Scuola Superiore Sant’Anna, 56127 Pisa, Italy; vito.monaco@santannapisa.it; 2 Scuola di Ingegneria, Università di Pisa, 56126 Pisa, Italy; c.giustinoni@studenti.unipi.it; 3 IRCCS Fondazione Don Carlo Gnocchi, 20148 Milan, Italy; tciapetti@dongnocchi.it (T.C.); amaselli@dongnocchi.it (A.M.); 4 Healthcare Engineering Innovation Center (HEIC), Khalifa University, Abu Dhabi P.O. Box 127788, United Arab Emirates

**Keywords:** breathing, IMUs, optoelectronic plethysmography, respiratory rate, tidal volume, accuracy

## Abstract

This study aimed to explore novel inertial measurement unit (IMU)-based strategies to estimate respiratory parameters in healthy adults lying on a bed while breathing normally. During the experimental sessions, the kinematics of the chest wall were contemporaneously collected through both a network of 9 IMUs and a set of 45 uniformly distributed reflective markers. All inertial kinematics were analyzed to identify a minimum set of signals and IMUs whose linear combination best matched the tidal volume measured by optoelectronic plethysmography. The resulting models were finally tuned and validated through a leave-one-out cross-validation approach to assess the extent to which they could accurately estimate a set of respiratory parameters related to three trunk compartments. The adopted methodological approach allowed us to identify two different models. The first, referred to as *Model 1*, relies on the 3D acceleration measured by three IMUs located on the abdominal compartment and on the lower costal margin. The second, referred to as *Model 2*, relies on only one component of the acceleration measured by two IMUs located on the abdominal compartment. Both models can accurately estimate the respiratory rate (relative error < 1.5%). Conversely, the duration of the respiratory phases and the tidal volume can be more accurately assessed by *Model 2* (relative error < 5%) and *Model 1* (relative error < 5%), respectively. We further discuss possible approaches to overcome limitations and improve the overall accuracy of the proposed approach.

## 1. Introduction

The development of wearable technology for the assessment of respiratory activity has grown in the last decades due to the broad range of available sensors [1,2,3,4]. Lightweight sensors and electronics embedded in customized gear (e.g., pants and *t*-shirts) or accessories (e.g., belts and bras) are designed to gather relevant information from the users to monitor their respiratory activity and recognize early signs of abnormal physiological conditions, such as cardiac or respiratory arrest [4], chronic respiratory diseases [5,6], or other psycho-physiological stressors [7].

Several recent review studies provide an overview of the currently available wearable technology to assess the most relevant features of respiratory mechanics. Among those reviews, Dinh and colleagues provided an up-to-date summary of stretchable respiration sensors based on different sensing principles [1]. The authors noted that the integration of sensors with other electronics and power sources into a single wearable device represents acurrent challenge that needs to be addressed. In addition, they noted that additional effort is required to improve the stretchability and wearability of respiration sensors without causing performance degradation. Soon and colleagues provided an overview of devices that are currently available on the market and designed for the remote monitoring of vital signs, including respiration, particularly in outpatient settings [3]. These authors, in accordance with other ones [4], noted that the routine use of these devices, in both outpatient and healthcare settings, is still limited. In addition, they pointed out that the literature lacks solid evidence to verify the effectiveness of some of the reviewed products. Massaroni and colleagues focused their attention on contact-based methods for measuring the respiratory rate [7]. The authors identified a wide taxonomy of the available technology and provided a detailed analysis of their metrological properties, characteristics, and potential applications. Finally, in one of our recent works, we collected research that supports the use of wearable devices to continuously monitor tidal volume in both daily activities and clinical settings [8]. We concluded that some of the proposed methodological approaches (i.e., optical, resistive, and inductive stretch sensors) have been widely investigated in the literature, while others are at an early stage. However, in both cases, the accuracy of the proposed wearable solutions might not be sufficient. Overall, current evidence does support the use of wearable technology to monitor respiratory activity, particularly in subjects affected by respiratory diseases outside the clinical setting. However, additional effort is required to overcome the limits documented in earlier reports.

The use of inertial measurement units (IMUs) has become popular in all branches of human biomechanics, such as in sports [9,10], clinical applications [11,12], biomedical engineering [13,14], and virtual reality [15,16]. These sensors are typically composed of a multi-axial accelerometer and a multi-axial gyroscope; thus, they are sensitive to the linear acceleration and angular velocity of the body segment they are attached to. During the last two decades, several studies have investigated the accuracy of different algorithms, parsing the output (acceleration and/or angular velocity) of one or more IMUs to assess respiratory activity as well.

Authors have adopted a single dual- or tri-axial accelerometer as an inclinometer to reconstruct the slow angular movement of either the chest or the abdomen and to extract some of the features (i.e., respiratory rate and tidal volume) related to respiratory activity [17,18,19]. Others determined the respiratory-related variation in the trunk orientation by fusing signals from a tri-axial accelerometer and a tri-axial gyroscope by means of a Kalman filter [20,21,22,23]. These approaches focus on the variation in the trunk orientation determined by the respiratory activity based on the evidence that the respiratory-related magnitude of the inertial acceleration is relatively small compared to a change in the gravitational components. Other authors analyzed the frequency content of signals from a single dual- or tri-axial accelerometer located on either the chest or the abdomen to estimate the respiratory rate [24,25,26]. Fekr and colleagues adopted a similar sensory setup (i.e., one tri-axial accelerometer located on the sternum) to reconstruct respiratory patterns and, accordingly, recognize respiratory disorders [27,28]. Two studies dealt with the use of either two tri-axial accelerometers or two tri-axial IMUs located on the anterior and posterior chest walls [29,30]. By using differential approaches, these authors estimated the respiratory-related chest expansion by reducing the effects of the translational movements of the user, particularly during dynamic motor tasks. Finally, De la Fuente and colleagues adopted a network of 13tri-axial accelerometers to develop an automatic strategy revealing costal-superior and costal-abdominal respiratory patterns [31].

Based on the current literature, the main strengths of an IMU-based approach to monitoring respiratory activity are: (i). the low cost of sensors that are widely available on the market; (ii). their low power consumption, allowing for a long lifespan between charges; (iii). the inherent lightweight and small dimensions of hardware modules embedding sensors, a microcontroller, power supply, and memory and/or wireless data transmission technologies, which make these devices unobtrusive and potentially powerful for human wellbeing monitoring during daily activities; and iv. the respiratory activity assessment through IMUs can be considered one of the most convenient and noninvasive strategies to monitor subjects, such as children, who lack cooperation when undergoing mildly invasive approaches, such as spirometry.

Despite the mentioned, though non-exhaustive, array of studies dealing with IMU-based approaches to monitoring respiratory activity, a wide variability can be found among them. This variability mainly refers to the adopted sensors (e.g., accelerometers vs. gyroscopes), the sensory location (e.g., chest vs. abdomen and sternum vs. left costal margin), the algorithms for the assessment of the most relevant features of breathing patterns (e.g., trigonometric vs. Kalman filter-based data fusion), and the application-related approach (e.g., static vs. dynamic motor tasks and single vs. differential sensory strategy). In addition, some of the proposed strategies have been roughly validated, so the outcomes of these studies appear to be quite preliminary [7,8].

The aim of this study was to evaluate the feasibility of an IMU-based strategy to estimate respiratory patterns in subjects lying on a bed. In this respect, our study was designed to overcome some of the main methodological limits of the current literature (e.g., limited number of enrolled subjects, subject-specific tuning of algorithms, and different experimental setups) to achieve robust results that unequivocally support or reject the use of IMUs for the estimation of respiratory patterns. In addition, since our results corroborated the former hypothesis (i.e., feasibility of an IMU-based strategy to estimate respiratory patterns), the accuracy that we achieved with the proposed approach represents a benchmark to compare other algorithms. In the long term, we would like to contribute to the development of an alternative, noninvasive methodology to monitor significant variations in respiratory activity (e.g., changes in lung elasticity and progression of chronic respiratory diseases) in bedridden patients.

To achieve this multifaceted goal, our work was structured in two steps. During the first step, we analyzed the kinematics captured by a network of 9IMUs and 45 reflective markers equally distributed on the anterior wall of the trunk of subjects lying on a bed (Section 2.1,Section 2.2 and Section 2.3). Then, we estimated the respiratory patterns by means of optoelectronic plethysmography and used them as reference variables (Section 2.4). After that, through principal component analysis (PCA), we searched for the minimal set of signals coming from the network of IMUs where there was a match with linear combinations for the respiratory patterns of the whole chest wall, the rib cage compartment, and the abdominal compartment as a function of time (Section 2.5). This last task was based on the evidence that signals collected by an IMU as a function of time likely follow chest movements during respiratory activity [17,18,19]. The second step consisted in developing a model relating the retained minimal set of signals coming from the network of IMUs to respiratory patterns. This model was finally validated by using the leave-one-out cross-validation (LOOCV) procedure (Section 2.6). Notably, the adopted two-step strategy was designed to prevent any significant effect of anatomical dimorphisms due to gender and subject-specific features on the accuracy of the estimates.

## 2. Materials and Methods

### 2.1. Enrolled Participants

Eighteen healthy young adults were enrolled in this study. Participants had no history of diseases (e.g., respiratory, postural, or neurological) that could interfere with the experimental sessions. Age and anthropometrical features are reported in Table 1. All participants signed an informed consent form before starting the experimental sessions.

### 2.2. Experimental Setup and Protocol

**Figure 1:**
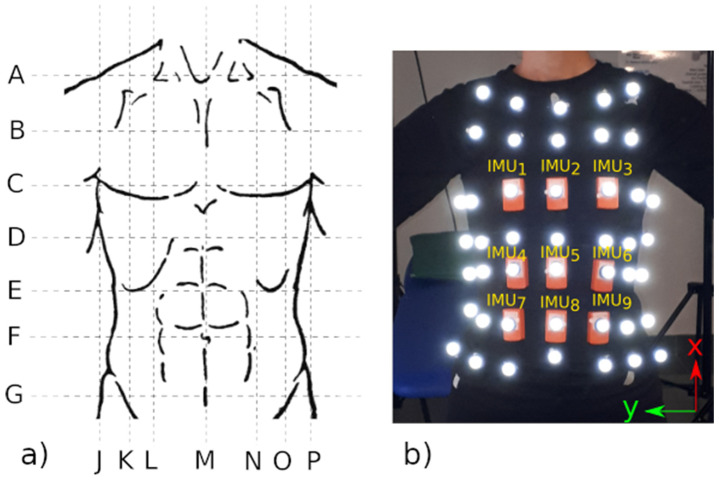
The left panel (**a**) shows the grid adopted to uniformly span the anterior trunk wall with reflective markers. Horizontal lines are craniocaudally identified as follows: A connects the lateral ends of both clavicles, B is equally distant from A and C, C connects the nipples, D crosses the xiphoid process, E connects the lower costal margins, F crosses the umbilicus, and G connects the anterior iliac spines. Vertical lines are identified as follows: K and O cross the right and left anterior iliac spines, respectively; M is the midline crossing the umbilicus; L and N cross the nipples; and J and Pare right and left midaxillary lines, respectively. The right panel (**b**) shows the adopted marker set. Note that markers are located on the nodes of the grid shown in the left panel except for those related to line A, which are positioned along the clavicles. The panel also shows the location of the nine IMUs (orange boxes) and their orientation (see x- and y-axes in the bottom right corner).

The participants were asked to lie supine on a bed at rest while the respiratory movements of the chest and abdomen were contemporaneously recorded by an optoelectronic system (8-camera optical system Smart-DX, BTS Bioengineering, Milano, Italy) and nine IMUs (Xsens wireless Motion Tracker Awinda system [32]), as depicted in Figure 1. A set of 45 reflective markers (diameter of 10 mm) were located on the anterior wall of the trunk, from the collarbone to the segment connecting the anterior iliac spines. The location of the marker set likely resembled that reported in earlier studies dealing with optoelectronic plethysmography [33,34,35]. Nine of the markers were attached in the case of the nine IMUs (Figure 1). The axes of all IMUs were oriented as follows: the x-axis ran longitudinally, towards the head; the y-axis was the latero-lateral axis, towards the right side; and the z-axis was arranged according to the right-hand role (vertical, upward orientated). Data collected by the optoelectronic system and the IMUs were digitalized at 50 Hz and 100 Hz, respectively.

During the experimental sessions, participants were asked to breathe normally fora 2 min time window. At the beginning of each trial, IMU_1_ (Figure 1) was delicately beat about three times to induce quasi-impulsive variations in the kinematics measured by the optoelectronic system and the inertial sensors. These impulsive signals were used for offline synchronization of both data streams, as described elsewhere [36] and in Section 2.3. For each participant, data related to 2 repetitions were collected. The time gap between the repetitions was about 2 min. Overall, we collected 36 pairs of data streams (i.e., 18 subjects × 2 repetitions).

The study was conducted in accordance with the Declaration of Helsinki, and the protocol was approved by the Ethics Committee of the Scuola Superiore Sant’Anna, Pisa (IT) (protocol code: 01/2021; date of approval: 11 February 2021).

### 2.3. Pre-Processing

Both the 3D kinematics of the 45 reflective markers and the output of each of the 9IMUs (3 linear accelerations and 3 angular velocities) were pre-processed offline according to the following pipeline: i. gap filling, if any; and ii. zero-lag, low-pass filtering (Butterworth, 4th order) with cut-off at 10 Hz. Notably, although the literature agrees on the evidence that most of the power spectral density of chest motion kinematics while breathing normally falls within 1.5–2 Hz [18,27,30], we decided to account for a wider frequency bandwidth, i.e., 0–10 Hz, to prevent any lack of information that could negatively affect the outcome of the PCA.

After that, spline interpolation was applied to the 3D kinematics of the reflective markers to virtually double its sample rate and match that of the IMUs (i.e., 100 Hz). This step was required to minimize the error associated with the estimated time lag during the time alignment procedure.

Data from the camera system and the IMUs were time-aligned by assessing the time lag between them. Specifically, we computed the cross-correlation between the second time derivative of the vertical component of the marker on IMU_1_ and the Z-component of the acceleration detected by the same IMU within a time window including the quasi-impulsive artifacts induced by the beats. The time lag between the data streams coincided with the abscissa of the maximum of their related cross-correlation function.

### 2.4. Assessing the Tidal Volume as a Function of Time by Optoelectronic Plethysmography

The approach to estimating tidal volume as a function of time based on the 3D kinematics of the trunk, namely, optoelectronic plethysmography, is widely described in earlier literature [33,34]. Briefly, this method consists of approximating the chest wall in a series of non-overlapped triangles in which the vertexes coincide with three neighboring markers. Then, the frame-by-frame variation in chest wall volume, namely, V_CW_, is computed as the summed contribution of all volumes enclosed by these triangles.

With respect to our study, we assumed that the respiratory activity was mostly reflected in the movement of the frontal view of the chest, since the posterior wall was constrained due to the subject’s supine position on the bed. In addition, we assumed that the bias due to the thickness of the IMUs (~13 mm) on the vertical component of the related markers did not substantially alter the tidal volume estimation. Then, based on our marker set, we defined 64 non-overlapped triangles that were symmetrically arranged with respect to the midline (vertical line M in Figure 1). After that, V_CW_ was estimated as the volume enclosed by all these triangles and split with respect to the rib cage (where V_RC_ is the volume enclosed between lines A and E) and the abdomen (where V_AB_ is the volume enclosed between lines E and G) compartments.

### 2.5. Principal Component Analysis (PCA) of IMU Outputs

The PCA was run to reduce the dimensionality of the input dataset consisting of the output from the IMUs. In particular, it was carried out to identify a subset of IMU-related homologous signals (i.e., either accelerations or angular velocities) where a linear combination allowed for an optimal match with V_CW_, V_RC_, and V_AB_.

For each subject and each trial, we initially defined eight data matrices by pooling homologous signals related to all IMUs as follows:

(1) $$A_{X}=[A_{X}^{1},A_{X}^{2},\ldots ,A_{X}^{9}],$$

(2) $$A_{Y}=[A_{Y}^{1},A_{Y}^{2},\ldots ,A_{Y}^{9}],$$

(3) $$A_{Z}=[A_{Z}^{1},A_{Z}^{2},\ldots ,A_{Z}^{9}],$$

(4) $$A_{ToT}=[A_{ToT}^{1},A_{ToT}^{2},\ldots ,A_{ToT}^{9}],$$

(5) $$\Omega_{X}=[\Omega_{X}^{1},\Omega_{X}^{2},\ldots ,\Omega_{X}^{9}],$$

(6) $$\Omega_{Y}=[\Omega_{Y}^{1},\Omega_{Y}^{2},\ldots ,\Omega_{Y}^{9}],$$

(7) $$\Omega_{Z}=[\Omega_{Z}^{1},\Omega_{Z}^{2},\ldots ,\Omega_{Z}^{9}]$$

(8) $$\Omega_{ToT}=[\Omega_{ToT}^{1},\Omega_{ToT}^{2},\ldots ,\Omega_{ToT}^{9}]$$

where:

- $A_{i}^{j}$ and $\Omega_{i}^{j}$ represent the *i*th component (i.e., X, Y, or Z components) of acceleration and angular velocity, respectively, measured by the *j*th IMU (*j* = 1, 2, …, 9);
- $A_{ToT}^{j}$ and $\Omega_{ToT}^{j}$ represent the Euclidean norm of the three components of the acceleration and angular velocity, respectively, related to the *j*th IMU.

**Figure 2:**
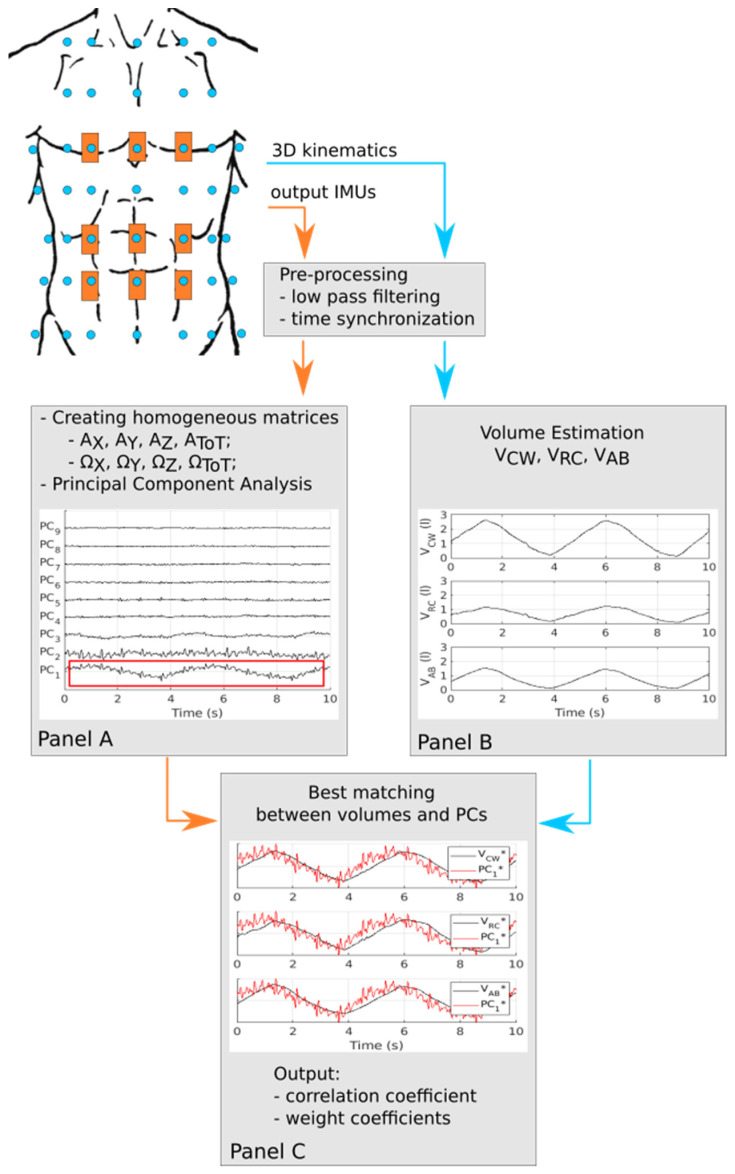
Summary of the algorithm that we used to identify the principal components (PCs) that best matched the time course of respiratory volumes (i.e., V_CW_, V_RC_, and V_AB_). For this representative example, all data refer to the first repetition of subject #5. In addition, data reported in Panel (**A**) were computed from matrix A_X_. The red box in Panel (**A**) identifies the PC that best matches (in this representative case) respiratory volumes according to the Pearson correlation coefficient. The volumes shown in Panel (**B**) are shifted along the vertical axis to set their minimum values in the whole data stream to 0. The valleys and peaks refer to onset of the inhalation and exhalation phases, respectively. The data reported in Panel (**C**) were z-scored (see apex * in the legend) to make the comparison straightforward.

Each of these eight data matrices was first z-scored and then parsed by the PCA. Briefly, the PCA projects the initial dataset (e.g., $A_{X}$, $A_{Y}$, $A_{Z}$, and $A_{ToT}$) in a new domain of orthogonal (i.e., uncorrelated) variables, namely, the principal components (PCs), through a squared weight coefficient data matrix reflecting the degree of association between the initial data and PCs [37]. Next, we retained the PCs that had the best match with V_CW_, V_RC_, and V_AB_ according to the Pearson correlation coefficient (ρ). The whole process is described in Figure 2.

After that, we pooled data among the subjects and trials and identified the subset of IMUs sharing more variance with the retained best-matching PCs, i.e., those more correlated with respiratory patterns (i.e., V_CW_, V_RC_, and V_AB_). Specifically, we assumed that the outcome of an IMU was significantly associated with one of the retained best-matching PCs when the related absolute weight coefficient, as averaged across participants and trials, was ≥0.32. This threshold implies that the observed variable (i.e., output of the IMU) shared more than 10% of its variance with that PC [37].

Finally, for all subjects and all trials, the signals related to the selected subset of IMUs and the V_CW_, V_RC_, and V_AB_ values as a function of time were low-pass filtered (zero-lag, Butterworth, and 4th order) with a cut-off at 2.5 Hz, demeaned, and used to develop and validate a model relating IMU outputs to tidal volumes.

### 2.6. Developing IMU-Based Models for Tidal Volume Estimation

In this study, we assumed that a linear combination of signals collected by a minimal set of IMUs can accurately estimate respiratory volumes, in accordance with previous findings [17,18,19]. We adopted the LOOCV strategy to test the ability of the designed model to predict new data that were not used to tune the model itself [38]. According to LOOCV, datasets (i.e., both volumes and retained IMUs outputs) related to 17 out of 18 subjects were selected to train the model. The training process consisted in finding optimal *n*-tuples of model coefficients that minimized the residuum between the measured and estimated volumes across those subjects and throughout the entire time window. After that, the optimal *n*-tuples of the model coefficients were used to estimate the respiratory volumes related to the 18th subject for both repetitions.

For both the measured and estimated volumes, we identified the relative minima and maxima within the entire time window, which represented the onsets of the inhalation and exhalation phases, respectively. Based on these time events, the respiratory rate (where RR is the number of respiratory cycles per minute), the duration of the inhalation and exhalation phases (DI and DE, respectively), and the inhaled and exhaled volumes (VI and VE, respectively) for the chest wall, ribcage, and abdomen compartments were computed and averaged across cycles. Finally, the relative error between measured and estimated respiratory variables (i.e., ΔRR%, ΔDI%, ΔDE%, ΔVI%, and ΔVE%) was computed and analyzed across conditions (see Section 2.7).

The next paragraphs provide a detailed description of both the algorithm that we used to develop the model relating the tidal volume to the output of the retained IMUs and the strategy that we used to validate it through the LOOCV. For the sake of simplicity, the following sections report the procedure adopted for the estimation of V_CW_ only. The same procedure was separately implemented for V_RC_ and V_AB_.

The relationship between the *estimated* chest wall volume and signals collected by the minimal set of IMUs (outcome of the PCA) is represented by the following equation:

(9) $${\tilde{V}}_{CW}\left(t\right)=m\cdot \left(k_{1}^{CW}\cdot {\mathrm{var}}_{1}\left(t\right)+k_{2}^{CW}\cdot {\mathrm{var}}_{2}\left(t\right)+\ldots +k_{n}^{CW}\cdot {\mathrm{var}}_{n}\left(t\right)\right)$$

where:

- m refers to the body mass of the subject and was introduced to account for the monotonic relationship between body mass and volume capacity [39,40];
- t refers to the time;
- ${\tilde{V}}_{CW}\left(t\right)$ is the *estimated* chest wall volume as a function of time;
- var_1_(t), var_2_(t), and var_n_(t) represent the *n*-tuple of signals collected by the retained minimal set of IMUs as the outcome of the PCA, where these variables are subject-dependent;
- k_1_^CW^, k_2_^CW^, and k_n_^CW^, namely, the model coefficients, are the coefficients of the linear model, where the training procedure consisted of tuning these coefficients.

A system of 34 (i.e., 2 repetitions × 17 subjects) non-linear equations was then formulated as follows:

(10) $$RMSD^{i,j}=rms\left(V_{CW}^{i,j}\left(t\right)-{\tilde{V}}_{CW}^{i,j}\left(t\right)\right)$$

where:

- $V_{CW}^{i,j}\left(t\right)$ and ${\tilde{V}}_{CW}^{i,j}\left(t\right)$ are the measured and estimated V_CW_, respectively, for the *i*th subject and *j*th repetition;
- RMSD^i,j^ is the root mean square (rms) of the difference between the measured and estimated V_CW_ for the *i*th subject and *j*th repetition.

After that, a minimum search algorithm (see fminsearch in Matlab) was run to find the optimal *n*-tuple for the model coefficients (i.e., k_1_^CW^, k_2_^CW^, …, and k_n_^CW^) that minimized the norm of the vector [RMSD^1,1^, RMSD^1,2^, …, RMSD^17,1^, and RMSD^17,2^].

Once the optimal *n*-tuple for the model coefficients (i.e., k_1_^CW^, k_2_^CW^, …, and k_n_^CW^) was found, it was used to estimate the volume of the 18th subject for both repetitions. This process was complemented by a comparison of respiratory parameters (i.e., the respiratory rate and inhalation and exhalation durations and volumes) between the values obtained through optoelectronic plethysmography and those estimated by the model in terms of relative error percentage (i.e., ΔRR%, ΔDI%, ΔDE%, ΔVI%, and ΔVE%).

### 2.7. Statistical Analysis

The mean and standard deviation were used as the main descriptive statistics to refer to the central tendency and dispersion of all independent variables (i.e., age and anthropometrical features of the enrolled participants, outcomes of the PCA analysis, respiratory variables, and errors). After that, inferential statistics, especially unpaired *t*-test and analysis of variance (ANOVA) with repeated measures, were used to investigate the effects of gender, IMU-related outputs, compartments, and models on all independent variables. Notably, when the data distribution of independent variables did not meet the requirements for a normal distribution (Kolmogorov–Smirnov normality test) and homogeneity of variance (Levene’s test), these statistical tests were carried out on the Log_10_ transformed data.

The significance for all statistical tests was set at *p* < 0.05. The data analysis was carried out using Matlab (The MathWorks, Inc., Natick, MA, USA).

## 3. Results

The enrolled participants consisted of 7 females and 11 males with a comparable age between groups, although, as expected, the males were taller and heavier than their female counterparts (Table 1).

### 3.1. Outcomes of the PCA

#### 3.1.1. Identification of Homologous Data Matrices That Best Match PCs with the Respiratory Volumes

The outcomes of the PCA in terms of the correlation coefficients between the best-matching retained PCs and respiratory volumes (i.e., V_CW_, V_RC_, and V_AB_) are reported in Table 2, Table 3 and Table 4, respectively. The results revealed that the PCs obtained by parsing acceleration matrices (i.e., A_X_, A_Y_, A_Z_, and A_ToT_) were well correlated (ρ > 0.71) with V_CW_, V_RC_, and V_AB_. Conversely, the correlations between volumes and PCs related to the angular velocity matrices (i.e., Ω_X_, Ω_Y_, Ω_Z_, and Ω_ToT_) were typically poor (ρ < 0.43). Notably, no differences between the groups of subjects (i.e., F vs. M) were observed.

Based on these findings, we investigated possible differences in terms of ρ among the acceleration data matrices (i.e., A_X_, A_Y_, A_Z_, and A_ToT_) only (the angular velocity data matrices were excluded from further analysis since they correlated poorly with the respiratory volumes, as shown in Table 2, Table 3 and Table 4). This analysis was undertaken to identify which among the homologous acceleration data matrices (i.e., A_X_, A_Y_, A_Z_, and A_ToT_) more accurately reproduced the respiratory patterns. The outcome of one-way ANOVA with repeated measures revealed that the distribution of ρ reported in Table 2, Table 3 and Table 4 was significantly (*p* < 0.001) different across the acceleration data matrices (i.e., A_X_, A_Y_, A_Z_, and A_ToT_). In this respect, Tukey’s post hoc test highlighted that ρ obtained by processing A_Z_ was lower than the values related to the other acceleration data matrices (i.e., A_X_, A_Y_, and A_ToT_).

**Figure 3:**
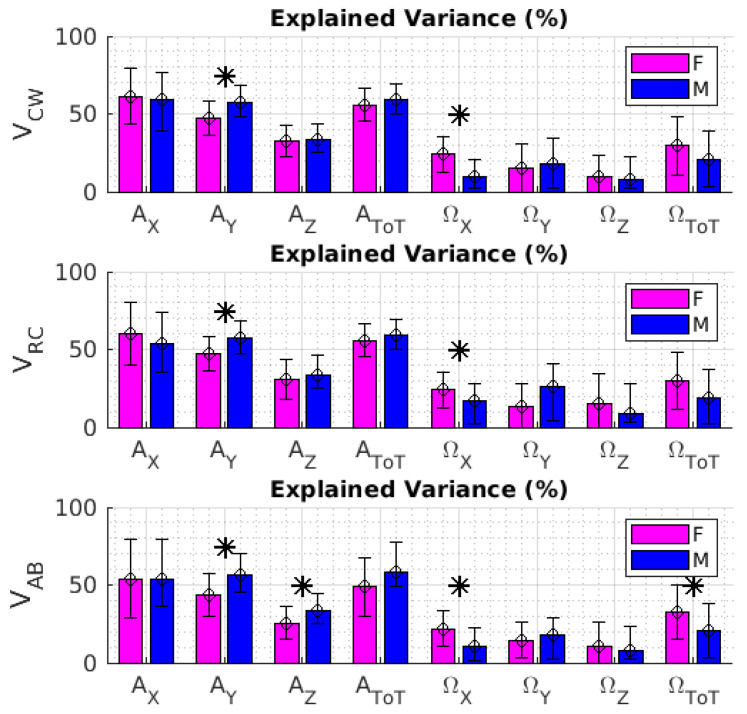
The three panels depict the variance explained by the best-matching retained PCs related to volumes V_CW_, V_RC_, and V_AB_, respectively. Bars and error bars respectively refer to mean and standard deviation of the explained variance across subjects. Data reported in magenta and blue refer to male and female. The label * represents the outcome of the unpaired *t*-test when significant (i.e., *p* < 0.05).

The variance explained by the best-matching retained PCs greatly changed based on the input data matrix and, in some cases, with respect to gender (Figure 3). Specifically, the explained variance was higher for the acceleration data matrices (i.e., A_X_, A_Y_, A_Z_, and A_ToT_) than for the data matrices accounting for angular velocity (i.e., Ω_X_, Ω_Y_, Ω_Z_, and Ω_ToT_). Specifically, for the acceleration data matrices (i.e., A_X_, A_Y_, A_Z_, and A_ToT_), the explained variance averaged across subjects was typically close to or greater than 50% for A_X_, A_Y_, and A_ToT_, while it dropped to approximately 30% or less for A_Z_. For Ω_X_, Ω_Y_, Ω_Z_, and Ω_ToT_, the explained data variance was, on average, always lower than 32%. For a limited set of data matrices, we also observed significant differences between females and males (*p* < 0.05 for the outcome of the paired *t*-test).

To summarize, the outcome of the PCA on the IMU outputs revealed that the linear accelerations as a function of time, especially those referring to A_X_, A_Y_, and A_ToT_, had the best associations with the respiratory patterns related to all compartments (i.e., V_CW_, V_RC_, and V_AB_) assessed by ρ (Table 2, Table 3 and Table 4). In this respect, the data variance explained by the best-matching retained PCs was, on average, about 50% (Figure 3).

#### 3.1.2. Selecting the Minimum Set of IMUs

**Figure 4:**
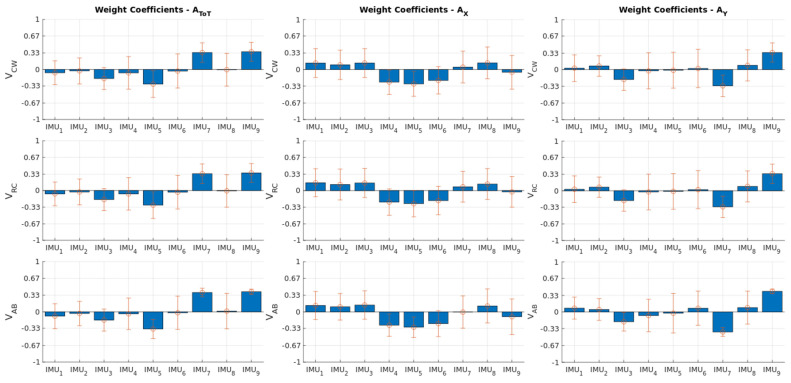
The subplots show the weight coefficients relating IMU outputs (from left to right: homologous data matrices A_ToT_, A_X_, and A_Y_) to retained PCs that best match with respiratory patterns (from top to bottom: V_CW_, V_RC_, and V_AB_). The bars and error bars refer to the averaged and standard deviations across subjects, respectively.

To define the minimal set of IMUs, we analyzed the weight coefficients relating the best-matching retained PCs to the IMU outputs and identified those ≥0.32. The results reported in Figure 4 highlight a significant association between both V_CW_ and V_RC_ and the norm of the 3D acceleration measured by IMU_7_ and IMU_9_. In addition, a significant association was also observed between V_AB_ and the norm of 3D acceleration assessed by IMU_5_, IMU_7_, and IMU_9_. Alternatively, V_AB_, V_CW_, and V_RC_ were significantly associated with the Y-component of the acceleration measured by IMU_7_ and IMU_9_.

Concerning the weight coefficients obtained after parsing A_X_, the results showed that their average across subjects was always below the threshold (i.e., 0.32), suggesting that the X-component of the acceleration of all IMUs on average shared less than 10% of the variance with the retained PCs (Figure 4).

Overall, the results revealed that two minimal sets of IMUs are expected to accurately estimate the respiratory patterns as a function of time. The first set accounted for two IMUs that were laterally located on the abdominal compartment (see IMU_7_ and IMU_9_ in Figure 1) and one IMU centrally located on the segment connecting the lower costal margins (see IMU_5_ in Figure 1). According to this configuration, the IMUs were expected to collect all acceleration components to finally estimate A_ToT_. The second set accounted for only two IMUs that were laterally located on the abdominal compartment (see IMU_7_ and IMU_9_ in Figure 1) and designed to collect the Y-component of the acceleration.

### 3.2. Model Validation

According to the outcomes of the previous analysis, two alternative IMU-based models appeared suitable to contemporaneously estimate the volumes related to all compartments (i.e., V_CW_, V_RC_, and V_AB_). The first model (*Model 1*) relies on the 3D acceleration measured by IMU_5_, IM_7_, and IMU_9_ as follows:

(11) $$\{\begin{array}{c} {\tilde{V}}_{CW}\left(t\right)=m\cdot \left(k_{7}^{CW}\cdot A_{ToT}^{7}\left(t\right)+k_{9}^{CW}\cdot A_{ToT}^{9}\left(t\right)\right)\\ {\tilde{V}}_{RC}\left(t\right)=m\cdot \left(k_{7}^{RC}\cdot A_{ToT}^{7}\left(t\right)+k_{9}^{RC}\cdot A_{ToT}^{9}\left(t\right)\right)\\ {\tilde{V}}_{AB}\left(t\right)=m\cdot \left(k_{5}^{AB}\cdot A_{ToT}^{5}\left(t\right)+k_{7}^{AB}\cdot A_{ToT}^{7}\left(t\right)+k_{9}^{AB}\cdot A_{ToT}^{9}\left(t\right)\right) \end{array}$$

where:

- m and t are body mass and time, respectively;
- $A_{ToT}^{5}$, $A_{ToT}^{7}$, and $A_{ToT}^{9}$ are the 3D acceleration measured by IMU_5_, IMU_7_, and IMU_9_, respectively; and
- $k_{7}^{CW}$, $k_{9}^{CW}$, $k_{7}^{RC}$, $k_{9}^{RC}$, $k_{5}^{AB}$, $k_{7}^{AB}$, and $k_{9}^{AB}$ are the model coefficients.

The second model (*Model 2*) relies on the Y-component of acceleration measured by IM_7_ and IMU_9_, as follows:

(12) $$\{\begin{array}{c} {\tilde{V}}_{CW}\left(t\right)=m\cdot \left(h_{7}^{CW}\cdot A_{Y}^{7}\left(t\right)+h_{9}^{CW}\cdot A_{Y}^{9}\left(t\right)\right)\\ {\tilde{V}}_{RC}\left(t\right)=m\cdot \left(h_{7}^{RC}\cdot A_{Y}^{7}\left(t\right)+h_{9}^{RC}\cdot A_{Y}^{9}\left(t\right)\right)\\ {\tilde{V}}_{AB}\left(t\right)=m\cdot \left(h_{7}^{AB}\cdot A_{Y}^{7}\left(t\right)+h_{9}^{AB}\cdot A_{Y}^{9}\left(t\right)\right) \end{array}$$

where:

- m and t are body mass and time, respectively;
- $A_{Y}^{7}$ and $A_{Y}^{9}$ are the Y-components of the acceleration measured by IMU_7_ and IMU_9_, respectively;
- $h_{7}^{CW}$, $h_{9}^{CW}$, $h_{7}^{RC}$, $h_{9}^{RC}$, $h_{7}^{AB}$, and $h_{9}^{AB}$ are the model coefficients.

For both models, we analyzed the accuracy of the estimation of the five respiratory parameters (i.e., RR, DI, DE, VI, and VE) extracted from V_CW_, V_RC_, and V_AB_ as a function of time.

#### Analysis of Accuracy

**Figure 5:**
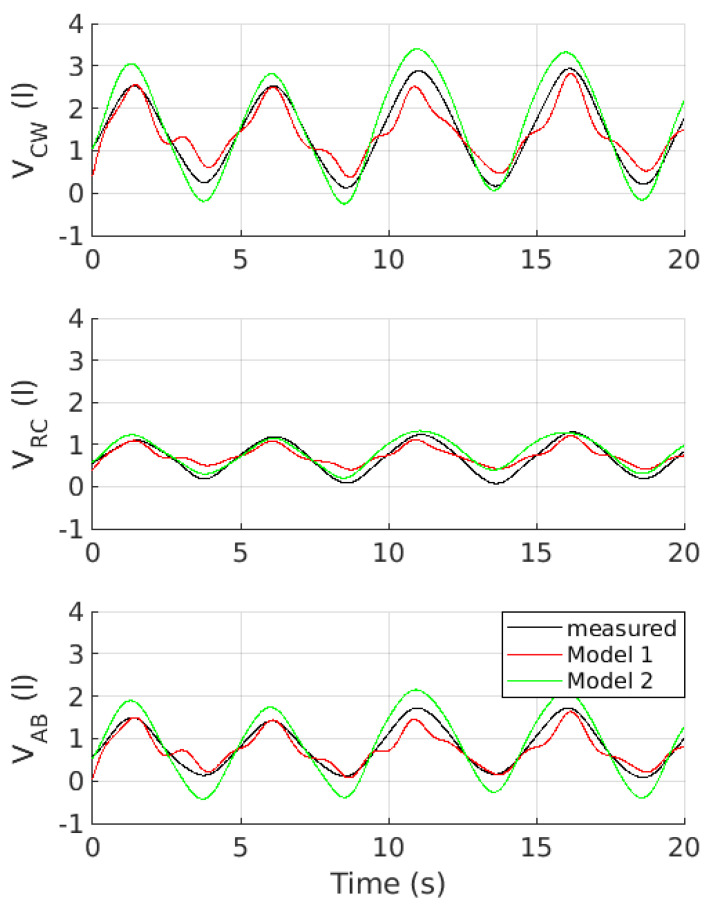
From top to bottom, the panels show a representative example of the tidal volume measured by optoelectronic plethysmography (**black curves**) and estimated by both *Model 1* (**red curves**) and *Model 2* (**green curves**) and related to the chest wall (V_CW_), rib cage (V_RC_), and abdominal (V_AB_) compartments.

Figure 5 shows a representative example of V_CW_, V_RC_, and V_AB_ as a function of time measured by optoelectronic plethysmography and estimated by both *Model 1* and *Model 2*.

**Figure 6:**
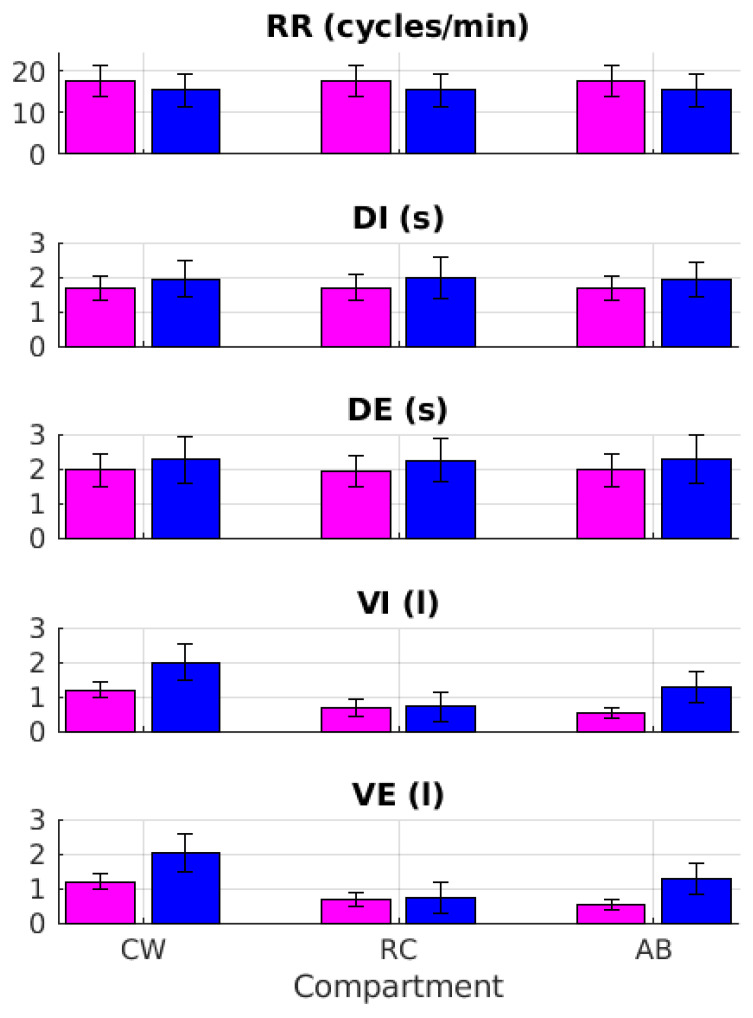
From top to bottom, the panels show mean and one standard deviation error bar for all respiratory variables (i.e., RR, DI, DE, VI, and VE), as measured by optoelectronic plethysmography, across compartments (i.e., CW, RC, and AB) for both female and male groups (pink and blue, respectively).

Figure 6 reports the average and data dispersion for all respiratory parameters grouped by compartment (i.e., V_CW_, V_RC_, and V_AB_) and gender (i.e., F and M) measured by optoelectronic plethysmography.

Two-way ANOVA with repeated measures was carried out to investigate the effects of gender (two levels: female and male) and compartment (three levels: CW, RC, and AB) on the respiratory parameters (i.e., RR, DI, DE, VI, and VE) measured by optoelectronic plethysmography (Figure 6). The results (Table 5) revealed that variables related to the respiratory timing (i.e., RR, DE, and DI) were not different between the groups or among the compartments. However, as expected, both factors significantly affected the volumes (factor for gender: *p*-value = 0.002; factor for compartments: *p*-value < 0.001). In particular, the values related to the inhalation and exhalation volumes were larger in males. In addition, volumes related to sub-compartments RC and AB were smaller than those observed for the whole CW compartment.

**Figure 7:**
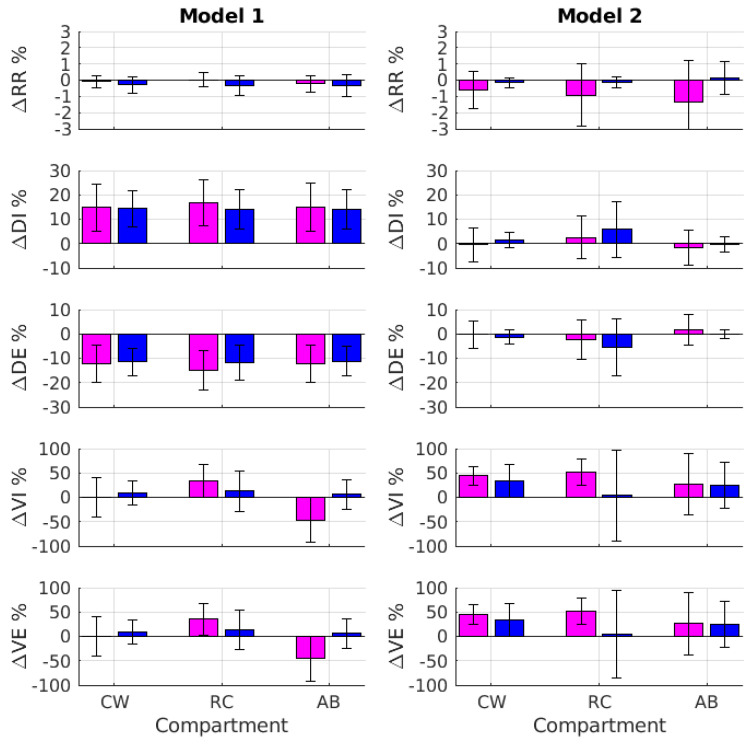
From top to bottom, the panels show the error percentage (mean and one standard deviation error bar) concerning all assessed respiratory variables (i.e., RR, DI, DE, VI, and VE) related to IMU-based *Model 1* (on the **left**) and *Model 2* (on the **right**) for both female and male groups (pink and blue, respectively).

Figure 7 shows the relative error percentage concerning all assessed respiratory variables (i.e., RR, DI, DE, VI, and VE) related to the IMU-based *Model 1* and *Model 2*.

A three-way ANOVA with repeated measures was carried out to investigate the effects of gender (two levels: female and male), compartment (three levels: CW, RC, and AB), and model (two levels: *Model 1* and *Model 2*) on the relative error percentages reported in Figure 7. The outcome of this test (Table 6) revealed that the relative error percentage related to RR was not significantly affected by the three factors (*p* > 0.05). In this respect, both IMU-based models accurately estimated the respiratory rate with an absolute relative error that was below 1.5% among groups and compartments when averaged across subjects. Notably, the gender factor did not significantly affect (*p* > 0.05) the relative error related to any of the respiratory variables (i.e., ΔRR%, ΔDI%, ΔDE%, ΔVI%, and ΔVE%).

On the other hand, the compartment and model factors significantly (*p* < 0.05) affected the relative error related to the estimated duration of both the inhalation and exhalation phases (i.e., ΔDI% and ΔDE%, as shown in Table 6). The values of ΔDI% and ΔDE% related to *Model 1* (CW: ΔDI% = 14.5 ± 8.2;ΔDE% = −11.9 ± 6.3; RC: ΔDI% = 15.0 ± 8.6; ΔDE% = −13.0 ± 7.6; AB: ΔDI% = 14.4 ± 8.7; and ΔDE% = −11.6 ± 6.7) were typically larger than those associated with *Model 2* (CW: ΔDI% = 0.7 ± 5.0;ΔDE% = −0.9 ± 4.2; RC: ΔDI% = 4.5 ± 10.4; ΔDE% = −4.2 ± 10.4; AB: ΔDI% = −0.8 ± 5.1; and ΔDE% = 0.6 ± 4.2). Moreover, the absolute values of ΔDI% and ΔDE% were smaller for the abdominal compartment and increased for the chest wall and the rib cage.

For the tidal volume estimation, the statistical analysis revealed that ΔVI% and ΔVE% were significantly (*p* < 0.05) affected by the model factor. Specifically, the accuracy of the relative error associated with *Model 1* was typically about seven times smaller than that related to *Model 2* (*Model 1*: ΔVI% = 3.9 ± 41.2 and ΔVE% = 4.3 ± 41.0.9; *Model 2*: ΔVI% = 28.7 ± 56.4 and ΔVE% = 28.7 ± 56.1).

## 4. Discussion

This study evaluated novel IMU-based strategies to estimate respiratory parameters in subjects lying on a bed while breathing normally.

Our approach consisted in contemporaneously collecting data from both a network of 9 IMUs and a set of 45 reflective markers uniformly distributed on the frontal chest wall (Figure 1). The inertial kinematics were initially parsed by PCA to identify them inimum set of signals and IMUs whose linear combination best matched the tidal volume measured by optoelectronic plethysmography. The results (Table 2, Table 3 and Table 4; Figure 3 and Figure 4) revealed that linear accelerations as a function of time related to two different subsets of IMUs had the best contemporaneous associations with tidal volumes related to the whole chest wall, rib cage, and abdominal compartments.

Based on these findings, two different models were developed: i. the first model, namely, *Model 1*, consisted of the linear combination of the 3D acceleration measured by two IMUs laterally located on the abdominal compartment (see IMU_7_ and IMU_9_ in Figure 1) and one IMU centrally located on the segment connecting lower costal margins (see IMU_5_ in Figure 1); ii. the second model, namely, *Model 2*, consisted of the linear combination of the Y-component of two accelerometers symmetrically located on the abdominal compartment (see IMU_7_ and IMU_9_ in Figure 1). The models were assumed to depend on the body mass of the subjects. They were finally tuned and validated through a LOOCV approach to assess the extent to which subject-independent models can accurately estimate five respiratory parameters (i.e., RR, DI, DE, VI, and VE) related to three trunk compartments (i.e., the whole chest wall, rib cage, and abdominal).

The results revealed that both models accurately estimated the respiratory rate with an average relative error lower than 1.5% across subjects and conditions (Figure 7). The duration of the inhalation and exhalation phases (i.e., DI and DE) was more accurately assessed by Model 2, where the amplitude of the average relative error across subjects was lower than 5% for all compartments (Figure 7). Finally, the values of the tidal volume during the inhalation and exhalation phases were more accurately estimated by *Model 1*. In this case, the absolute relative error averaged across subjects was lower than 5%, with no difference among the compartments (Figure 7 and Table 6).

### 4.1. Achieved Accuracy

Several studies tested IMU-based strategies to assess respiratory parameters under comparable experimental conditions. Certain authors estimated the respiratory rate from the output of a tri-axial accelerometer by using different approaches and reported either a relative error ranging from 0.53% [27,28] to 10% [26] or an absolute error falling in the range 0.38–0.50 breaths/min [18,24]. Other authors working with the output of a gyroscope reported an absolute error in the respiratory rate ranging from 0.3 to 7.9 breaths/min [21,22]. As far as the inhalation and exhalation volume estimation is concerned, earlier studies mainly showed a correlation coefficient between the measured tidal volume and that estimated through IMU-based methods (range: 0.77–0.96) as a function of time [18,27]. However, no information concerning breath-by-breath accuracy was reported.

Compared to results in the literature, our findings appear to be more accurate and may even be further improved. The accuracy underlying the estimation of RR obtained in our study (mean relative error equal to 1.5%, roughly corresponding to 0.24 ± 0.06 breaths/min) can be considered comparable to or even better than that reported in previous papers [21,22,26,27,28]. In addition, the error related to the chest wall tidal volume estimation, which was lower than 5% on average, is equivalent to that achieved by the most accurate wearable-based strategies reviewed in our recent study [8]. In our opinion, this performance can be further improved by adopting suitable expedients. For instance, it is worth noting that wearable-based strategies for the assessment of respiratory parameters typically rely on a subject-specific calibration procedure. Conversely, we evaluated the feasibility of subject-independent strategies to assess the main respiratory parameters. In this respect, we envisage that a subject-specific tuning of the models can result in improved accuracy for the estimation of respiratory parameters. Moreover, the PCA-based approach that we used to identify a minimal set of signals and IMUs can potentially represent another factor limiting the overall accuracy of the developed models. Other data reduction strategies (e.g., factor analysis) or decomposition procedures (e.g., wavelet decomposition) in conjunction with a non-linear combination of IMU signals may allow for the development of more accurate models that match the tidal volume as a function of time. Overall, the accuracy that we achieved when estimating RR can be considered acceptable, but that related to the tidal volume is not yet in the range of ±3.5%, which has been recommended for calibration checks during spirometry [41]. However, some of the weak points of our methodological strategy can be strengthened, and this strategy deserves to be further investigated to verify whether the overall accuracy can be improved.

### 4.2. Comparison of Model 1 and Model 2

One of the unexpected outcomes of our study was the identification of two alternative models to contemporaneously estimate respiratory activity among trunk compartments: the first relies on the 3D acceleration measured by three IMUs (i.e., IMU_5_, IMU_7_, and IMU_9_ in Figure 1), and the second relies on the Y-component only of the acceleration measured by two IMUs located on the abdominal compartment (i.e., IMU_7_ and IMU_9_). It is worth remarking that there is a significant overlap between them; that is, both models mostly rely on the respiratory-related movements of the abdominal compartment. This result corroborates findings in the literature, where the authors documented a significant association between respiratory movements of the abdominal compartment and the supine position [42,43]. As such, others concluded that abdominal movements can be used to represent diaphragmatic excursion in a healthy population while lying supine [35].

*Model 1* resulted in a more accurate estimation of the tidal volume, whereas *Model 2* provided a more accurate estimation of the duration of the inhalation and exhalation phases (Figure 7 and Table 6). This evidence suggests that rib cage movements, in conjunction with a more informative set of signals due to the 3D components of acceleration, can better fit the tidal volume across compartments, as highlighted by *Model 1* (i.e., V_CW_, V_RC_, and V_AB_; see Figure 7). However, the 3D components of acceleration and the physiological phase shift between rib cage and abdominal movements [44] would result in a superimposition of components with a different frequency content (compare *Model 1* and *Model 2* in Figure 5) that involve a fictive shift in either the peaks or valleys of the tidal volume as a function of time. Accordingly, the onset of either the inhalation or exhalation phases in *Model 1* would artificially appear to be premature or delayed. In turn, the duration of both the inhalation and exhalation phases would be reciprocally biased (see Figure 7, *Model 1*, ΔDI% and ΔDE%).

### 4.3. Limitations of This Study

The main limitation of our study is that the models that we developed to estimate the respiratory parameters during normal breathing in a supine position cannot be generalized to either different motor tasks or breath patterns. The performance of IMU-based strategies to assess the main respiratory parameters decreases during dynamic tasks (e.g., walking, running, and cycling) due to body motion artifacts [7,8]. In addition, pathological breath patterns (e.g., bradypnea, tachypnea, Cheyne–Stokes, Kussmaul, and Biot’s) can be characterized by arrhythmias or different movements of the chest wall compared to normal breathing. Accordingly, in both cases, we envisage that the final models that we used to estimate respiratory parameters might not be optimal to accurately assess respiratory parameters in different experimental conditions. Despite this, the approach that we used to identify the minimal set of IMUs and how to relate their output to respiratory activity deserves to be fully investigated and validated across different experimental conditions.

## 5. Conclusions

Our work suggests that the estimation of respiratory parameters for subjects in a supine position can be accurately carried out by using a limited set of sensors mainly located on the abdominal compartment and, if needed, on the lower costal margin. It is worth remarking that the approach that we used to develop and validate IMU-based models was quite conservative. Accordingly, we envisage that the achieved accuracy can be further improved.

We also observed that different sensor configurations (i.e., two IMUs vs. three IMUs) allowed for the contemporaneous estimation of respiratory parameters among trunk compartments with different accuracies. Although the sensory network incorporating three IMUs may represent a suitable approach to take advantage of both the models, we believe that the final design of any IMU-based system aimed at accurately assessing respiratory activity should consider both the need to contemporaneously gather information among all trunk compartments and other features, particularly those related to wearability and appearance. In this respect, further research is required to identify an optimal compromise between the accuracy of the measurement tool and usability.

## Figures and Tables

**Table 1:** Age and anthropometrical features of enrolled participants (mean ± standard deviation). The *p*-value is the outcome of the unpaired *t*-test comparing the effect of the factor gender on the independent variables. The *p*-values are highlighted in bold when reaching significance (*p* < 0.05).

	7 Female	11 Males	*p*-Value
Age (years)	32.7 ± 7.7	31.3 ± 7.3	0.696
Body mass (kg)	57.3 ± 9.0	74.0 ± 11.4	**0.005**
Height (m)	1.66 ± 0.05	1.76 ± 0.06	**0.002**

**Table 2:** Pearson correlation coefficient between best-matching retained PCs and V_CW_ (mean ± standard deviation). The *p*-value is the outcome of the unpaired *t*-test comparing the effect of the factor gender on the independent variables. The *p*-values are highlighted in bold when reaching significance (*p* < 0.05).

Input Data Matrix	Female	Males	*p*-Value
A_X_	0.87 ± 0.10	0.85 ± 0.09	0.707
A_Y_	0.86 ± 0.12	0.93 ± 0.06	0.086
A_Z_	0.75 ± 0.11	0.80 ± 0.09	0.210
A_ToT_	0.90 ± 0.09	0.92 ± 0.05	0.443
Ω_X_	0.38 ± 0.13	0.32 ± 0.13	0.191
Ω_Y_	0.36 ± 0.13	0.36 ± 0.09	0.914
Ω_Z_	0.27 ± 0.10	0.29 ± 0.06	0.480
Ω_ToT_	0.43 ± 0.11	0.39 ± 0.07	0.254

**Table 3:** Pearson correlation coefficient between best-matching retained PCs and V_RC_ (mean ± standard deviation). The *p*-value is the outcome of the unpaired *t*-test comparing the effect of the factor gender on the independent variables. The *p*-values are highlighted in bold when reaching significance (*p* < 0.05).

Input Data Matrix	Female	Male	*p*-Value
A_X_	0.84 ± 0.11	0.78 ± 0.09	0.124
A_Y_	0.85 ± 0.12	0.83 ± 0.0.8	0.607
A_Z_	0.71 ± 0.15	0.71 ± 0.12	0.870
A_ToT_	0.86 ± 0.12	0.83 ± 0.09	0.395
Ω_X_	0.37 ± 0.14	0.30 ± 0.13	0.201
Ω_Y_	0.33 ± 0.14	0.36 ± 0.08	0.626
Ω_Z_	0.26 ± 0.09	0.26 ± 0.06	0.788
Ω_ToT_	0.41 ± 0.10	0.37 ± 0.07	0.204

**Table 4:** Pearson correlation coefficient between best-matching retained PCs and V_AB_ (mean ± standard deviation). The *p*-value is the outcome of the unpaired *t*-test comparing the effect of the factor gender on the independent variables. The *p*-values are highlighted in bold when reaching significance (*p* < 0.05).

Input Data Matrix	Female	Male	*p*-Value
A_X_	0.84 ± 0.13	0.86 ± 0.09	0.534
A_Y_	0.87 ± 0.11	0.93 ± 0.08	0.084
A_Z_	0.74 ± 0.11	0.86 ± 0.08	0.050
A_ToT_	0.89 ± 0.14	0.93 ± 0.07	0.297
Ω_X_	0.38 ± 0.11	0.33 ±0.12	0.204
Ω_Y_	0.39 ± 0.10	0.36 ± 0.09	0.394
Ω_Z_	0.27 ± 0.11	0.30 ± 0.06	0.397
Ω_ToT_	0.42 ± 0.10	0.39 ± 0.07	0.380

**Table 5:** Outcomes of two-way ANOVA for the analysis of gender (2 levels: female and male) and compartment (3 levels: CW, RC, and AB) on the respiratory parameters measured by optoelectronic plethysmography (data reported in Figure 6). The *p*-values are highlighted in bold when reaching significance (*p* < 0.05).

*p*-Values/Factor	Gender	Compartment
RR	0.259	0.990
DI	0.277	0.145
DE	0.285	0.633
VI	**0.002**	**<0.001**
VE	**0.002**	**<0.001**

**Table 6:** Outcomes of three-way ANOVA for the analysis of gender (2 levels: female and male), compartment (3 levels: CW, RC, and AB), and model (2 levels: *Model* 1 and *Model 2*) on the relative error percentage of respiratory parameters assessed with IMU-based models (data reported in Figure 7). The *p*-values are highlighted in bold when reaching significance (*p* < 0.05).

*p*-Values/Factor	Gender	Compartment	Model
ΔRR%	0.209	0.808	0.179
ΔDI%	0.881	**0.026**	**<0.001**
ΔDE%	0.939	**0.008**	**<0.001**
ΔVI%	0.470	0.267	**<0.001**
ΔVE%	0.487	0.280	**<0.001**

## Data Availability

Not applicable.
